# P-558. Leveraging Interactive SMS Training to Equip Health Workers for Inclusive HIV Services Amid Uganda's Anti-Gay Law Implementation

**DOI:** 10.1093/ofid/ofaf695.773

**Published:** 2026-01-11

**Authors:** Christine Rwabyogamu Nabukera

**Affiliations:** Infectious Diseases Initiative, Ministry of Health, Kampala, Kampala, Uganda

## Abstract

**Background:**

In May 2023, Uganda passed an anti-homosexuality law. The law imposes the death penalty for men who have sex with men (MSM) and prohibits healthcare practitioners from providing HIV prevention, treatment, and care services to them. Consequently, many health workers lived in fear and refrained from offering these services to suspected MSM.

The Infectious Diseases Program (IDP), a community project, advocates for the provision of equitable healthcare services without discrimination. In the past, IDP developed innovative approaches to mass training for resource-constrained communities. In response to the challenges posed by the law, IDP introduced an interactive SMS-based training program for health workers. SMS training proved to be a viable solution due to its extensive reach, compatibility with basic mobile phones, lack of dependence on internet connectivity, and user-friendly interface.

**Methods:**

In July 2023, IDP piloted a three-week course titled "Enhancing Access to Safe and Equitable HIV Services for Key Populations." The course covered modules such as HIV prevention approaches, addressing HIV-related stigma and discrimination, and creating friendly healthcare environments. The pilot program was conducted with 141 health workers in Wakiso District. Participants received module content three times a week, along with SMS quizzes featuring multiple-choice questions at the end of each module. Health workers submitted their answers through a toll-free platform that provided immediate feedback on whether their responses were correct or incorrect.

**Results:**

Of the health workers, 78% (82 females, 59 males) enrolled in the course and participated in the SMS quizzes. Among them, 81% provided correct answers, surpassing the 70% threshold required for earning certificates of completion. A prominent challenge that hindered participation was the demanding work schedules of health workers.

**Conclusion:**

The high percentage of participation and accuracy demonstrated the effectiveness of SMS-based training as a mass education approach for health workers in resource-limited settings. Building on this success, IDP scaled up the SMS training program to three additional districts and plans to assess its impact further.

**Disclosures:**

All Authors: No reported disclosures

